# Pain Management for Total Knee Arthroplasty: Single-Injection Femoral Nerve Block versus Local Infiltration Analgesia

**DOI:** 10.5812/ircmj.13247

**Published:** 2014-01-05

**Authors:** Mehdi Moghtadaei, Hossein Farahini, Seyed Hamid-Reza Faiz, Farzam Mokarami, Saeid Safari

**Affiliations:** 1 Department of Orthopedic Surgery, Rasoul Akram Hospital, Iran University of Medical Sciences, Tehran, IR Iran; 2 Department of Anesthesiology and Pain Medicine, Rasoul Akram Hospital, Iran University of Medical Sciences, Tehran, IR Iran

**Keywords:** Femoral Nerve Block, Analgesia, Morphine, Local Infiltration Analgesia

## Abstract

**Background:**

Pain is one of the major concerns of patients underwent Total Knee Arthroplasty (TKA); appropriate pain management is a key factor in patient's early physical fitness to move, physiotherapy, and most importantly, patient satisfaction.

**Objectives:**

In this study the analgesic effect of single injection femoral nerve block (SFNB) was compared with local infiltration analgesia (LIA).

**Patients and Methods:**

Forty patients who underwent TKA under spinal anesthesia were randomized to receive single femoral nerve block (group F) or intra-periarticular infiltration (group I). Group F received single injection 20cc ropivacaine (10mg/cc) and in group I, a combination of 300mg ropivacaine, 30mg ketorolac and 0.5mg epinephrine diluted to a volume of 150cc and locally injected in and around the knee joint in 3 stages. Postoperative pain intensity measured by Visual Analog Scale (VAS). Morphine consumption, mobilization time and patients’ satisfaction evaluated as well.

**Results:**

Group I had significantly lower morphine consumption in the first postoperative day (10 vs. 12.5mg, P-value < 0.05). Within 6 hours postoperatively, VAS score was statistically lower in group I compared to group F (3 vs. 4, P-value < 0.05). However, within 12 hours it was statistically higher in group I than group F (6 vs. 5, P-value < 0.05). Other parameters were not statistically different in two groups.

**Conclusions:**

Both methods LIA and SFNB provided excellent pain relief and lower morphine consumption following TKA. LIA is a surgeon-controlled analgesic technique, which can be used to enhance patients’ satisfaction and reduce the pain in the very early postoperative period by surgeon independently.

## 1. Background

As life expectancy increases, the number of total knee arthroplasty (TKA) has also increased in order to improve life quality and mobility (1). The number of TKA has surged from 5.5/1000 to 8.7/1000 in the US and and continues to show an increscent slope (2). TKA postoperative pain is severe and excruciating; it does not reduce from 48 to 72 hours after operation(3). Adequate pain relief has a considerable role in patients’ recovery, early start of physiotherapy and reducing hospital length of stay. It also eventually results in reduced risk of postoperative side-effects such as thromboembolism and hospital infections (4, 5).

Various methods have been examined and used to control TKA postoperative pain; simple methods like oral NSAIDs and intravenous opioid administration, newer techniques like epidural block and femoral nerve block in two forms ,single injection and continuous infusion (24 - 72 hours) of anesthetic with catheter or the most recent analgesia methods such as local infiltration analgesia (LIA) (1-3, 5, 6); are constantly evolving.

Taking opioids leads to notable side-effects such as nausea, vomiting, pruritus, drowsiness and urinary retention (7). These may have negative effects on the early start of physiotherapy (8). Taking a lot of NSAIDs increases GI bleeding risk. Epidural analgesia is another common method. Although, its pain relief effect is better than other mentioned methods, it has some disadvantages such as motor block, delay in physiotherapy, involvement of both knees (the operated and healthy knee) and postponing taking anticoagulant considering the risk of epidural hematoma, hypotension and urinary retention (9). Like the epidural block, femoral nerve block has also been proven to reduce morphine consumption (9-13). Considering its lack of motor block, lack of side effects mentioned for epidural analgesia and simplicity of procedure, femoral nerve block is one of the preferred methods to control TKA postoperative pain (1). Since only the anterior and medial part of the knee receives sensory nerves from femoral nerve, femoral nerve block cannot achieve complete pain control in itself, especially when the posterior compartments of the knee are manipulated (probably in cruciate-sacrificing prosthesis). Recent studies have evaluated the use of LIA in combination with a number of different drugs and the results regarding reduced morphine consumption and early mobilization after TKA have been encouraging (14).

In the previous studies contradictory results regarding the comparison of LIA and femoral nerve block have been reported. Although some studies reported femoral nerve block as more efficient in range of motion (ROM) and pain control postoperatively (15), many other studies have indicated LIA as a better technique (16, 17).

Most of studies carried out so far have used catheter to infuse the anesthetic (24 to 48 hours) and the comparisons have been made on this basis. Using catheter is more expensive, requires follow-up and has probable side effects and infections.

## 2. Objectives

This prospective, double- blind, randomized, : clinical trial designed to compare the quality of analgesia offered by single injection femoral nerve block (SFNB) and LIA and their effects on morphine consumption, patients' satisfaction, pain control and physical rehabilitation postoperatively.

## 3. Patients and Methods

The present study is a randomized, double- blind clinical trial according to the ethical principles of Helsinki Declaration and approved by the Ethical Committee of Iran University of Medical Sciences (Project number: 92/130/96). All the patients signed the written, informed consent of the study procedure. During January to June 2013, the study included 40 inpatients from orthopedic ward in Rasoul Akram Hospital, Tehran, Iran.

### 3.1. Inclusion and Exclusion Criteria

Forty patients with osteoarthritis scheduled for TKA were screened for eligibility. The inclusion criteria were: age 20 to 85 years old, American Society of Anesthesiologists (ASA) physical status classification between (I-III), and normal preoperative mobility. Exclusion criteria were patients with neuropathic pain or sensory disorders of the leg being operated, failed spinal anesthesia, therefore conversed to general anesthesia, a medical history showing previous operations on the suffering knee, allergy to the medicine used in the study, BMI > 40, diseases of kidney, heart or liver, joint inflammatory disease, chronic pain and disorders resulting in bleeding, such as GI bleeding. 

### 3.2. Sample Size and Randomization

**Figure 1.**
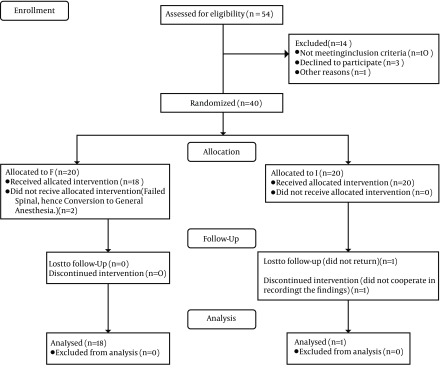
Consort Flow Diagram of Patients Through the Study

According to 40% difference in morphine consumption during the first 48 hours after surgery (regarding previous studies)(7), 80% strength, and coefficient α equal to 5% sample size of 36 patients were calculated. Considering the number of loss and withdrawal cases, 40 patients scheduled for TKA were enrolled and randomized, using a computerized blocked random number list, into two groups of 20 patients as SFNB (group F) or LIA (group I) (Figure 1). 

Patients, researchers, physiotherapists and all nursing staffs were blinded to the grouping. Only the surgeon was not blind to both groups and he had no role in later care of the patients.

### 3.3. Anesthesia and Analgesia Procedures

Premedication of 0.01 - 0.02 mg/kg midazolam was administered. Patients were placed in a lateral position. The drugs were injected by a 25-gauge Crawford needle in midline of L3 - L4 or L4 - L5 level by an anesthesiologist. For inducing spinal anesthesia, 15 mg of isobar intrathecal Marcaine® 0.5% (bupivacaine hydrochloride, 5 mg/mL, AstraZeneca Theatre Pack™, UK Limited) was administered without any additives. Then the patients were changed their position into supine and intranasal 100% oxygen was administered. Blood pressure less than 100 mmHg of 30% from the baseline was corrected by 5 mg ephedrine and crystalloids and all the pulse rate drops (less than 60/min) was treated with 0.5 mg intravenous atropine. Midazolam, ephedrine and atropine were provided from a regional pharmaceutical company (Darou-Pakhsh Co, Tehran, Iran).

All TKAs were carried out by medial parapatellar approach, A tourniquet was used for all patients and drain was removed on the day after surgery. Adhesive bandages were used in compressive form in a way that blocked venous and lymphatic channels.

In group I the injected substances included 300 mg ropivacaine 1% (Naropin, AstraZeneca, Sweden), 30 mg ketorolac (Syntex Laboratories, Palo Alto, CA) and 0.5 mg 1:100,000 epinephrine (Darou-Pakhsh Co, Tehran, Iran); of which 150 cc was prepared in 3 syringes (50 cc each).

**Figure 2.**
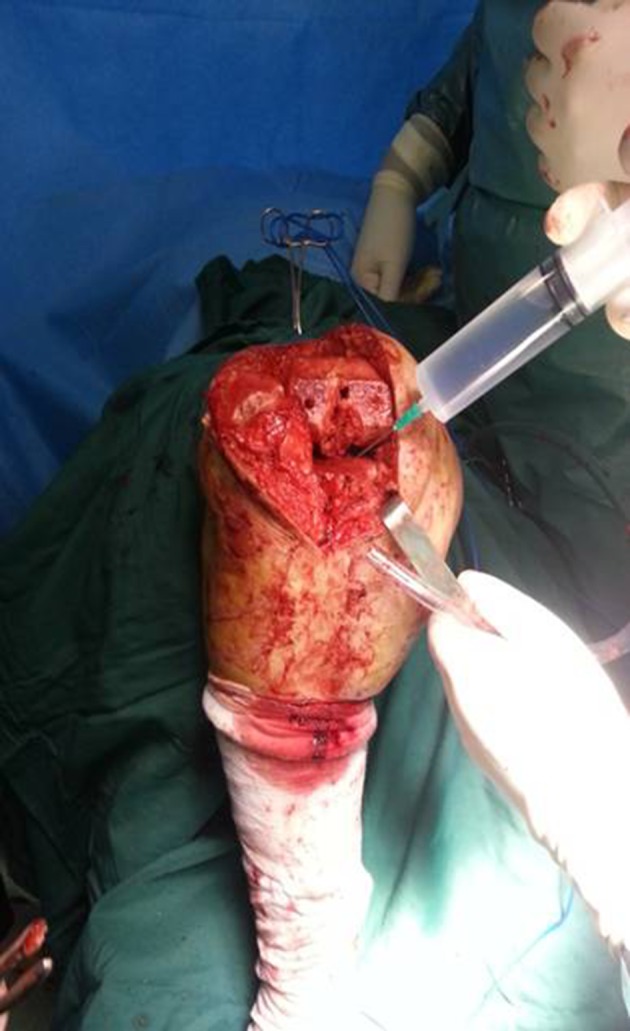
Injection Before Prosthesis Placement

**Figure 3.**
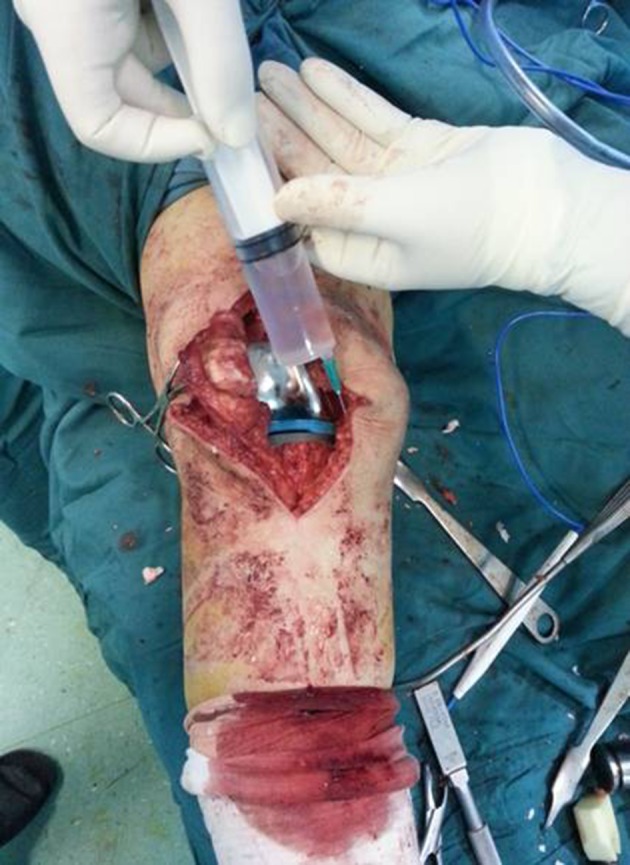
Injection After Prosthesis Placement

When the bone surface was prepared for the prosthesis to be placed, the first injection (50 cc of the mentioned solution) was done to the knee surrounding soft tissuefrom anterior to the posterior part and with 3 mm depth in the surrounding tissue (especially in the posterior capsule from one side to the other in a circular form). The second injection was made after placing the tibial and femoral components and again 50 cc was injected around the medial and lateral collateral ligament. The third injection (25 to 50 cc) was made before wound closure in the edges of the incision but deeper than subcutaneous tissue so that it prevents necrosis caused by epinephrine (Figures 2 and 3).

In group F a nerve stimulator was inserted1-1.5 cm lateral and inferior to the femoral artery, when the sensory level reached L1(inguinal area)in the recovery room setting until the quadriceps twitch at 0.5 mA could be seen. At this time 20 cc of ropivacaine 10 mg/cc was injected. In order to control pain in the first 48 hours, Acetaminophen 1 gr /BID orally, Ibuprofen 400 mg/ TDS orally and Ranitidine 50 mg/ BID (IV) were administered. The patients were instructed that no pain and worst possible pain equal to 0 and 10, respectively, on the visual analog scale (VAS). When patients’ VAS pain scores reach 4, morphine (5 mg) can be administered intravenously by nursing staff on the patients’ request (with minimum 1-hour intervals up to 6 times in 24 hours). Higher doses should be prescribed by physician order. Pain was controlled after 48 hours only with acetaminophen and oral tramadol.

### 3.4. Primary and Secondary Outcomes

In the present study, primary outcomes were morphine consumption 24 to 48 hours postoperatively, the VAS pain score, the patients’ satisfaction about the pain control in 48 hours postoperatively (excellent, good, satisfactory or poor), mobilization time which was the period between the operation and first walking experience (for 3 meters).

Secondary outcomes included side effects (nausea, vomiting, urinary retention, drowsiness, infection and convulsion), clinical outcome (compared with the measurement of knee flexion/extension at the time of patients’’ discharge and 3 months afterwards) and the number of hospitalization days (each night spent in hospital) after surgery. 

### 3.5. Data Analysis

Data were recorded in a questionnaire designed for the present study and Statistical Package for the Social Sciences (SPSS) software version 20 (SPSS Inc., Illinois, USA) was used to analyze the findings. Analysis of variables using Kolmogorov–Smirnov test showed that except ROM variable other variables did not follow normal distribution that is why Mann-Whitney U test was used instead of T-test to calculate P-values. Student’s T-test was used for the variables with normal distribution and Mann-Whitney U test was used for other parameters not normally distributed. Dichotomous data were analised using chi-square test or Fisher’s exact test. P < 0.05 was considered statistically significant.

## 4. Results

The flow diagram of patients through the study is shown in Figure 1. The patient characteristics and clinical data are summarized in Table 1. 

**Table 1.** Patients Characteristics Presented as mean ± SD for Variables

	Group F (N = 18)	Group I (N = 18)
**Sex (male/female)**	12/6	13/5
**Age, y**	67.4 ± 6.7	64 ± 6.9
**ASA ** ^**a**^ ** status I/ II/ III**	9/8/1	11/7/0
**Surgery time, min**	112 ± 11.4	115 ± 9.5
**Body Mass Index, kg m** ^**-2**^	28 ± 3	27 ± 2.5

^a^Abbreviation: ASA, American Society of Anesthesiologists

### 4.1. Morphine Consumption Amount

All the patients in both groups used morphine or oral tramadol to control pain within 48 hours postoperatively. After changing tramadol to its morphine equivalent (each 100 mg oral tramadol equals 10 mg IV morphine) it turned out that its consumption (10 mg) in group I in the first 24 hours after the surgery was significantly less than group F (12.5 mg) (P-value<0.05); however, there was no difference between the two groups within 48 hours after the surgery.

### 4.2. Postoperative Pain Score

Patients in group I suffered less pain (pain score 3) in the first 6 hours after the surgery compared with the patients in group F (pain score 4) but within 12 hours after the operation, group F reported less pain, and eventually 24 hours after the surgery the pain scores of the two groups did not demonstrate a significant difference (Table 2). 

**Table 2.** Pain Level, Morphine Consumption Level, Walking Time, Length of Stay and Patients’ Satisfaction Level

Variables	Group F ^d^	Group I ^d^	P-value
**Morphine consumption, mg**			
24 HPO ^**a**^	12.5 (10 - 20)	10 (5 - 10)	0.017
48 HPO	15 (13.75 - 25)	15 (10 - 20)	NS ^a^ 0.4
**VAS** ^**a**^			
6 HPO	4 (4 - 6)	3 (2 - 4)	0.002
12 HPO	5 (4.75 - 6)	6 (5 - 7)	0.024
24 HPO	6 (6 - 7)	6 (6 - 7)	NS (0.67)
**Time to Walk, h** ^**b**^	12 (11 - 12)	12 (10 - 24)	NS (0.88)
**Hospital Length of Stay, d**	5 (4 - 6)	5 (4.75 - 6.25)	NS (0.58)
**Satisfaction Level( after 48 hours)** ^**c**^	3 (2 - 3)	3 (2 - 3)	NS (0.56)

^d^Values are given as median with interquartile range in parentheses. Mann-Whitney U test

^a^Abbreviations: HPO, Hours Postoperation; NS, Not statistically significant; VAS, Visual Analog Scale

^b^ The time it takes for the patients to walk (at least 3 meters) after the surgery (in hours)

^c^ (1 = very good, 2 = good, 3 = satisfactory, 4 = poor)

### 4.3. Mobilization Time

Mobilization time is the time it takes for the patient to be able to walk at least 3 meters (with or without the help of physiotherapist) after the operation. No significant difference was observed between the two groups and a median of almost 12 hours was reached by each patient to first walk (Table 2). 

### 4.4. Side Effects

Group F reported side effects such as dizziness (2), nausea (1) and urinary retention (1). Also group I reported side effects; nausea (1), urinary retention (1) and dizziness (1). In addition one of the patients in group I suffered wound discharge continued for 4 weeks after the operation, no blood study and joint aspiration analysis and culture found positive for infection, however; the patient went under Irrigation and debridement and polyethylene exchange and the discharge resolved eventually. The two groups recorded no significant differences in side effects (P-value = 0.67).

### 4.5. Number of Hospitalization Days

Comparison of the number of hospitalization days (after surgery until showing appropriate conditions for discharge) in the two groups revealed no significant differences. Group F patients were hospitalized for 5.3 days and group I, 5.8 days on average (Table 2). 

### 4.6. The Patients’ Satisfaction with Pain Control

The satisfaction of the patients with the measures taken to control pain, did not reveal a significant difference between the two groups at the end of 48th hour after the surgery (P-value = 0.563) and the patients had described the result as “satisfactory”.

In group I the results were as follows; “good” (9), “satisfactory” (7) and “poor” (2) and group F reported 8 patients as “good” and 10 patients as “satisfactory” (Table 2). 

### 4.7. Knee Range of Motion after Knee Joint Replacement

Mean range of motion at the time of discharge was 66.9° in group F and 69.5° in group I. 3 months after the surgery, Mean range of motion was 112.2° in group F and 114.4° in group I. The results were not statistically significant (Table 3). 

**Table 3.** Knee Range of Motion

Variable	Group F^ a^	Group I^a^	P-value
**Knee Range Of Motion**			
**Discharge Day**	66.9 ° (9.9)	69.5 ° (8.9)	NS ^b^ 0.43
**3 months after the operation**	112.2 ° (14.4)	114.4 ° (11.5)	NS 0.61

^a^Values are Mean (SD).T- test has been used (normal distribution)

^b^Abbreviations: NS, Not statistically significant

## 5. Discussion

In the present study F and I methods were selected to be compared because previous studies had well indicated the effectiveness of these two methods in relieving pain after knee replacement surgery (1, 8, 18). The clinical outcomes have not demonstrated any significant differences in terms of ROM, Western Ontario and McMaster Universities Arthritis Index (WOMAC), Knee Society Evaluation and degree of physical activity (CHAMPS) in the follow-ups ranging from 3 months to 2 years with placebo-controlled group (15, 18, 19). However, a study showed that pain intensity after knee replacement surgery is the major concern of the patients volunteering for the operation and the pain relief methods with no extra effects will have the justification to be examined more. Despite the fact that methods using catheter offer better pain control compared with single injection methods (5, 17, 19, 20) they require more equipment and staff in the process of care after the operation and the most dangerous side-effect, namely infection, alarmingly accompanies the procedure. The occurrence of infections have been rarely reported in various studies (7, 21) but destructive effects of infection in arthroplasty can make it very difficult to justify even in a single case. In a study (22), femoral catheter was followed by superficial infection in 2 patients. In another study (23), although no infections were reported but bacterial colonization in 57% of patients was noted. In addition in other study (18) antibiotics were used until the time catheter was removed.

To our knowledge, there are few studies comparing single injection femoral nerve block with peri- and intra-articular methods. Ropivacaine dosage (300 mg) was derived from Ker and Kohan’s study (14) who pioneer in local infiltration analgesia, and most of such studies have been carried out by this dosage, though 400 mg ropivacaine also does not result in toxic serum levels (under 0.6 mg/mL) (9). Nevertheless, the exact approved nontoxic doses allowed into the joint are yet to be found out (24). Adding ketorolac seems reasonable to consider the trauma imposed on the healthy tissues and resulting inflammation.

Limitations of this study included converting palliative opioid amounts to their morphine equivalent which does not necessarily have to be the calculated doses, also utilizing a patient-controlled analgesia (PCA) set was not possible in this study and considering that a nurse was responsible to administer the opioid so another human factor affects the study. Double-blinding was strictly observed as far as possible, but paralyzed quadriceps muscle and numbness of the anterior thigh and medial side of the calf can distinguish group F from group I and have roles in administering more palliatives by the nurse. Considering the uprising of opioid receptors in the incision area due to inflammation and tissues trauma (17, 25), the addition of opioids to our 3-substance solution could be beneficial but it was not used due to the emphasis on the comparison of the two mostly accepted methods.

In the study at hand, morphine consumption in the first 24 hours after surgery in group I (10 mg) was less compared to group F (12.5 mg) which accords with the results of a study carried out by Karen Toftdah et al.(17). Also in other studies (16, 18) morphine consumption in group I was lower than placebo and epidural group in the first 24 hours.

Morphine consumption doses during the first 48 hours were not significantly different between the two groups which can be suggestive of the disappearance or decrease in the effectiveness of these two methods after 24 hours (P-value > 0.05). The VAS scores of the first 6 hours were lower in group I compared to group F; however, 12 hours after the surgery the patients in group F reported lower VAS scores (P-value < 0.05) that may stem from the hemobag drain (that was on for 10 minutes every 2 hours) and thus wash-out effect generated in method I. In this clinical trial walking time, side effects, patients’ satisfaction with pain control and the number of hospitalization days were not significantly different between the two groups (P-value > 0.05).

Also the clinical outcome (measured by ROM) did not show a significant difference between the two groups at the time of discharge and 3 months after the operation. These results accord with the findings of Won Sik Choy et al. (22)and Francis V. Salinas (19). This result does not seem unlikely considering the new rehabilitation protocols emphasizing ROM restoration in outclinic form (outpatients and patients outside the clinic) which bases the discharge criteria on the physical ability not pain control. According to Francis V. Salinas’s study mean hospitalization period is 4 days, the present study comes close to that figure with almost 5 days and reduce the role of pain control protocols in reducing length of stay.

The time it takes for both groups to walk for the first time after the surgery was almost 12 hours which shows no statistically significant difference (P-value = 0.89).Local infiltration analgesia (I) and femoral nerve block (F) methods are two appropriate methods to control TKA postoperative pain satisfactorily. Compared to femoral nerve block, local infiltration analgesia resulted in lower VAS scores in the first 6 hours and less morphine consumption in the first 24 hours after the surgery. It is also a surgeon-controlled analgesic technique; taking into account the fact that during the 24 to 48 hours of post-surgery all parameters showed not much difference and lack of statistically significant difference in the clinical outcome, hospitalization length and patients’ satisfaction level, with slight preference of local infiltration analgesia technique both methods can be used interchangeably depending on facilities and resources. It seems that pain control does not have a large effect on the improvement of final clinical outcome and length of stay. New physical therapy protocols (mostly outclinic) have diminished the role of pain control in achieving this goal. Fear of post-surgical pain is one of the most important concerns for the patients but by using (I) and (F) pain-relieving methods the fear can be dealt with and consequently patients’ satisfaction level can be improved to a great extent. We also recommend well-structured clinical trials to determine the dosage and kinds of other analgesic drugs with such protocols.
